# A Novel Residual Dual Attention Multiscale Network for Vibration-Based Damage Recognition in Floating Wind Turbine Structural Health Monitoring

**DOI:** 10.3390/s26134104

**Published:** 2026-06-28

**Authors:** Huiming Han, Yifei Li, Renqiang Wang, Hua Deng, Yuchen Lu, Yuxuan Zhang

**Affiliations:** 1 School of Nautical Technology, Jiangsu Maritime Institute, Nanjing 210024, China; 2 School of Engineering, Huzhou Normal University, Huzhou 313000, China; 3 Yantai Research Institute, Harbin Engineering University, Yantai 264006, China; 4 College of Intelligent Science and Engineering, Beijing University of Agriculture, Beijing 102206, China; 5 Department of Computer and Electrical Engineering, Mid Sweden University, SE-851 70 Sundsvall, Sweden

**Keywords:** floating wind turbine, structural health monitoring, vibration-based damage recognition, multi-scale feature extraction, attention mechanism

## Abstract

Floating wind turbines (FWTs) are key equipment for deep-sea clean energy exploitation, and their structural health condition is directly related to operational safety and energy output. However, FWT vibration signals exhibit significant non-stationary and multi-scale characteristics, with damage-sensitive features of different damage patterns spanning multiple temporal scales. Existing methods fail to sufficiently extract and fuse multi-scale damage-sensitive features. To this end, this paper proposes a novel Residual Dual Attention Multiscale Network (RDAMNet). The network innovatively designs a signal-level multi-scale decoupling strategy that extracts damage-sensitive features at different scales from complementary signal representations through a multi-branch differentiated architecture. Furthermore, an ECA-SE dual attention mechanism is designed to collaboratively enhance damage-related channel responses at both the feature extraction and fusion stages. Multiple independent experimental results on a publicly available dataset demonstrate that RDAMNet achieves a mean damage recognition accuracy and a weighted F1-score of 95.39% and 95.37%, respectively, significantly outperforming five compared methods. Cross-condition generalization experiments further demonstrate that RDAMNet maintains mean accuracies exceeding 94% across different wind speed and wind direction combinations, validating its stability across operating conditions. Moreover, RDAMNet only contains 663,783 parameters with a single-sample GPU inference time of 5.35 ms, exhibiting a favorable performance–efficiency trade-off. The ablation study verifies the effective contribution of each core component, and branch importance analysis, together with Grad-CAM visualization, further substantiates the multi-scale feature learning capability of the network. The proposed method provides an effective technical approach for intelligent structural health monitoring of FWTs in complex oceanic environments.

## 1. Introduction

Floating wind turbines (FWTs) serve as key equipment for deep-sea wind energy exploitation. They are capable of overcoming the water depth limitations of nearshore fixed-bottom wind turbines and harnessing stronger and more consistent wind resources in deep seas, thus playing an increasingly important role in the global clean energy transition and offshore renewable energy development [1,2,3,4,5,6,7,8]. However, FWTs operate over extended service periods in harsh oceanic environments characterized by high wind loading, intense wave excitation, salt spray corrosion, and continuous cyclic loading. These severe conditions subject critical structural components [9,10,11,12,13,14,15,16,17], including the tower, blades, floater connections, and mooring systems, to persistent degradation and damage risks. Early-stage structural damage, if not detected in a timely manner, may progressively propagate into severe structural failure under uncontrollable environmental loading, potentially leading to catastrophic consequences [18,19,20,21,22,23,24]. Given the highly restricted accessibility of offshore environments, manual inspection and maintenance operations are both costly and hazardous [25,26,27,28,29,30]. Therefore, developing automated structural health monitoring (SHM) techniques for FWTs is of paramount importance for enabling predictive maintenance, reducing unplanned downtime, and maximizing energy output [18,31,32,33,34,35,36].

Among the SHM techniques for FWTs, vibration-based methods have attracted extensive attention due to their broad applicability, cost-effectiveness, and capability for continuous real-time monitoring [37,38,39,40]. The core principle of this technique lies in the fact that structural damage induces measurable vibration response changes by altering stiffness, mass distribution, or damping properties [41,42,43,44,45]. In fact, vibration- and strain-response-based damage identification strategies have been extensively investigated for various engineering structures such as bridges, trusses, and pipelines [46,47,48,49,50], and these methods have accumulated a rich theoretical and methodological foundation for the field of structural damage identification. In terms of traditional machine learning, data-driven approaches leverage signal processing techniques to extract time-domain or frequency-domain features from vibration signals, combined with classifiers such as decision trees, artificial neural networks, k-nearest neighbors, and support vector machines for damage diagnosis [51,52,53,54]. Methods based on stochastic parametric models, such as autoregressive models and linear parameter-varying autoregressive models, achieve robust diagnosis of wind turbine structural damage through statistical testing. However, traditional machine learning methods rely on manual feature extraction and exhibit limited generalization capability in handling high-dimensional nonlinear data [55,56,57,58,59]. Deep learning has been widely adopted across multiple domains and continues to demonstrate outstanding performance, exhibiting strong automatic feature learning capability and superior generalization ability [60,61,62,63,64,65]. With the development of deep learning, convolutional neural networks, recurrent neural networks, and their hybrid architectures have shown significant advantages in wind turbine fault diagnosis, including end-to-end feature learning and improved generalization performance [66,67,68,69,70,71]. Meanwhile, attention mechanisms and multi-scale feature extraction strategies have been introduced to enhance the modeling of complex vibration patterns, and multisensory collaborative diagnosis frameworks have been further explored for damage localization and quantification in FWTs [72,73,74,75,76].

Although the aforementioned methods have achieved certain progress, existing research on FWT vibration-based damage recognition still suffers from several limitations. The vibration response signals of FWTs exhibit significant multi-scale time-frequency characteristics, and different types of damage manifest as differentiated signal signatures across varying temporal scales [77,78]. However, most existing methods employ single-scale or unified input representations for feature extraction, failing to sufficiently capture the complementary information among multi-scale damage-sensitive features. Moreover, the coupled effects of oceanic environmental noise and multi-source loading severely obscure early-stage subtle damage features [79,80]. Existing network architectures lack effective channel-level feature enhancement mechanisms, causing the responses of damage-sensitive channels to be diluted by noise-dominant channels. Meanwhile, current research on FWT vibration-based damage diagnosis predominantly focuses on mooring systems, with a notable lack of systematic investigation into early-stage damage recognition of other critical structural components, including the tower, floater connections, and blades.

To address these challenges, this paper proposes a novel Residual Dual Attention Multiscale Network (RDAMNet) for vibration-based damage recognition of FWTs. RDAMNet innovatively designs a signal-level multi-scale decoupling strategy that decomposes the raw vibration signal at the network front-end into complementary signal representations with distinct physical characteristics, and extracts damage-sensitive features at different scales through a differentiated branch architecture. Residual connections ensure the effective propagation of subtle damage features through deep networks. The ECA-SE dual attention mechanism collaboratively enhances damage-sensitive channel responses at both the feature extraction and fusion stages, effectively suppressing the interference of oceanic environmental noise. The proposed method is validated on a publicly available dataset. The main contributions of this paper are summarized as follows:

1. A novel Residual Dual Attention Multiscale Network (RDAMNet) is proposed for vibration-based damage recognition of FWTs, which innovatively designs a signal-level multi-scale decoupling strategy and a differentiated branch architecture to achieve complementary extraction of multi-scale damage-sensitive features, overcoming the limitation of existing methods in insufficiently capturing multi-scale information from unified input representations.
2. A dual attention mechanism composed of ECA and SE is designed to hierarchically enhance damage-sensitive channel responses at the feature extraction stage and adaptively recalibrate the contribution weights of cross-branch channels at the fusion stage, effectively mitigating the masking effect of oceanic environmental noise on subtle damage features.
3. Comprehensive experiments on the UPATRAS Floating Wind Turbine Vibration Dataset validate the effectiveness of RDAMNet, where multi-run comparative analyses, cross-condition generalization validation, ablation studies, and interpretability analyses systematically demonstrate the superiority of the proposed method from multiple perspectives, namely statistical reliability, generalization capability, component contribution, and feature visualization.

## 2. Proposed Method

### 2.1. Problem Formulation

Floating wind turbines (FWTs) operate in complex oceanic environments over extended service periods, and the vibration response variations induced by structural degradation serve as critical information sources for damage recognition. This study aims to leverage one-dimensional vibration signals acquired by nacelle-mounted accelerometers to automatically identify the structural health state of FWTs. The task is formulated as a supervised multi-pattern damage recognition problem. Given a one-dimensional vibration signal $x\in R^{L}$ of length *L*, the objective is to learn a mapping function $f:R^{L}\to \left\{1,2,\ldots ,C\right\}$ that assigns each signal to one of *C* predefined structural health states, where *C* denotes the total number of damage patterns including the healthy state. The training set $D={\left\{\left(x_{i},y_{i}\right)\right\}}_{i=1}^{N}$ comprises *N* labeled samples, where $y_{i}$ represents the ground-truth label of the *i*-th sample.

### 2.2. Overview of the Proposed RDAMNet

The vibration response signals of FWTs exhibit significant multi-scale time-frequency characteristics, and distinct damage patterns manifest as differentiated signal signatures across varying temporal scales. For instance, mooring line degradation induces low-frequency global stiffness variations that alter long-range structural responses, whereas localized structural defects such as crack propagation generate transient impulse signatures. Moreover, the coupled effects of oceanic environmental noise and multi-source loading further obscure damage-sensitive features. However, conventional single-branch convolutional neural networks, constrained by a fixed receptive field, fail to simultaneously capture both localized transient impulses and long-range structural variations. Meanwhile, standard convolutional operations assign equal weights across all feature channels, causing critical damage-related channel responses to be diluted by noise-dominant channels.

**Figure 1:**
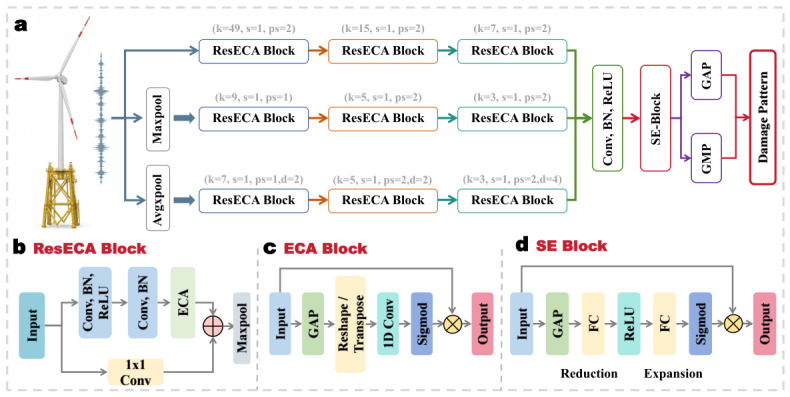
The overall architecture of the proposed RDAMNet.

To address these challenges, a novel Residual Dual Attention Multiscale Network (RDAMNet) is proposed for vibration-based damage recognition of FWTs. The core design philosophy of RDAMNet encompasses three aspects. Firstly, a multi-branch multi-scale input strategy is employed to extract complementary time-frequency features from the raw signal, max-pooled signal, and average-pooled signal, thereby enhancing the perceptual capability of the network across distinct damage patterns. Secondly, residual connections are incorporated within each feature extraction branch to mitigate the degradation of subtle damage features in deep networks, ensuring effective gradient propagation. Finally, a dual attention mechanism is introduced, which comprises ECA [81] and SE [82] attention. Specifically, ECA is embedded within each branch to enhance damage-sensitive channel responses, while SE operates on the post-fusion features for channel recalibration. The two mechanisms collaboratively achieve adaptive highlighting of damage-sensitive features. The overall architecture of the proposed RDAMNet is illustrated in Figure 1. To facilitate reproducibility, Table 1 lists the detailed configuration parameters of each module in RDAMNet and Table 2 further provides the internal specifications of the three core modules: ResECA, ECA, and SE.

The following subsections present each component of RDAMNet in the order of the data flow. The multi-scale signal input and differentiated branch design are introduced first, followed by the detailed structure of the ResECA feature extraction block within each branch. Subsequently, the principles of the ECA and SE attention mechanisms are described, respectively. Finally, the adaptive feature fusion and damage recognition output are presented.

### 2.3. Multi-Scale Signal Input and Differentiated Branch Design

In the vibration signals of FWTs, distinct types of damage signatures are distributed across varying temporal scales. Long-range low-frequency structural responses induced by global stiffness variations such as mooring line degradation, transient impulse signatures generated by localized defects such as crack propagation, and subtle damage features submerged in oceanic environmental noise each require extraction at different temporal resolutions. A single signal representation fails to simultaneously accommodate the extraction requirements of these multi-scale features. Therefore, RDAMNet applies max-pooling and average-pooling operations to the original input signal x at the network front-end, generating three complementary multi-scale input signals. Among them, the raw signal preserves the full time-domain resolution, the max-pooled signal retains the peak responses within local regions to highlight transient impulses and abrupt variations, and the average-pooled signal suppresses stochastic high-frequency noise to facilitate the extraction of stationary low-frequency structural responses. The three input signals can be formulated as:

(1) $$x_{1}=x,\;x_{2}={\mathrm{MaxPool}}_{p}\left(x\right),\;x_{3}={\mathrm{AvgPool}}_{p}\left(x\right)$$

where *p* denotes the pooling kernel size and stride, $x_{1}\in R^{1\times L}$ represents the raw signal, $x_{2}\in R^{1\times \lfloor L/p\rfloor }$ represents the max-pooled signal, and $x_{3}\in R^{1\times \lfloor L/p\rfloor }$ represents the average-pooled signal.

The three signals are fed into three differently configured feature extraction branches. Each branch comprises three cascaded ResECA feature extraction blocks, while employing differentiated configurations in terms of kernel size, pooling strategy, and dilation rate to match the physical characteristics of each signal.

The raw signal branch employs progressively decreasing large kernel sizes to extract low-frequency and global structural response features over extended temporal ranges. Larger convolutional kernels cover a wider temporal window, facilitating the capture of global vibration pattern variations induced by structural damage. The max-pooled branch employs smaller kernel sizes. Since the input signal has already been processed by max-pooling to retain peak information, this branch combined with small convolutional kernels is more effective at capturing localized impulse, abrupt variation, and spike response features. The average-pooled and dilated convolution branch introduces progressively increasing dilation rates on the basis of the average-pooled signal. The average-pooling operation suppresses stochastic noise beforehand, while dilated convolutions expand the receptive field without increasing the parameter count or further reducing temporal resolution, enabling this branch to capture structural response patterns spanning longer temporal extents under noise-suppressed conditions.

### 2.4. ResECA Feature Extraction Block

The vibration variations induced by early-stage structural damage in FWTs are often extremely subtle and tend to degrade or even vanish during the layer-by-layer abstraction process of multi-layer convolutions. To ensure these subtle damage features are effectively preserved in deep networks, the fundamental feature extraction unit within each branch adopts the ResECA structure. The residual connection constructs a shortcut path for identity mapping, enabling subtle damage features to be directly propagated across multiple convolutional layers to deeper levels. The ECA adaptively weights channel responses at each hierarchical level, highlighting damage-sensitive channels.

Specifically, this module comprises two layers of one-dimensional convolution, batch normalization (BN), ReLU activation, ECA channel attention, and a residual connection. Given the input feature $F_{\mathrm{in}}\in R^{C_{\mathrm{in}}\times T}$, the feature extraction process can be formulated as:

Specifically, this module comprises two layers of one-dimensional convolution [83], batch normalization (BN) [84], ReLU activation [85], ECA channel attention [81], and a residual connection. Given the input feature $F_{\mathrm{in}}\in R^{C_{\mathrm{in}}\times T}$, the feature extraction process can be formulated as

(2) $$H={\mathrm{Conv}1d}_{2}\left(\mathrm{ReLU}\left({\mathrm{BN}}_{1}\left({\mathrm{Conv}1d}_{1}\left(F_{\mathrm{in}}\right)\right)\right)\right)$$

(3) $$F_{\mathrm{out}}=\mathrm{Pool}\left(\mathrm{ReLU}\left(\mathrm{ECA}\left({\mathrm{BN}}_{2}\left(H\right)\right)+S\left(F_{\mathrm{in}}\right)\right)\right)$$

where S(·) denotes the shortcut connection, which is an identity mapping when the input and output dimensions are identical, or a 1×1 convolution with batch normalization when a dimensional transformation is required. Pool(·) represents the optional max-pooling operation for temporal downsampling.

### 2.5. Efficient Channel Attention

**Figure 2:**
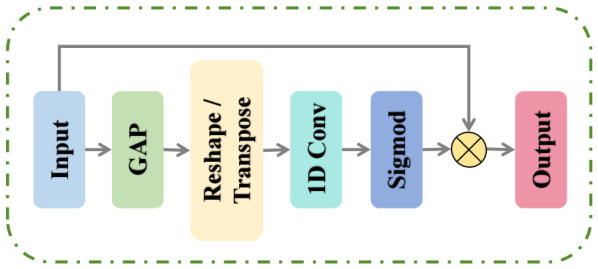
The principle of the ECA attention mechanism.

ECA is a lightweight channel attention mechanism proposed by Wang et al. [81]. Unlike SE attention that employs fully connected layers to model global channel relationships, ECA leverages one-dimensional convolution to achieve local cross-channel interaction, maintaining efficient channel modeling capability while avoiding the information loss caused by dimensionality reduction. RDAMNet embeds ECA within each ResECA block to adaptively enhance damage-sensitive channel responses at each hierarchical level. The principle of ECA is illustrated in Figure 2.

Given the input feature $F\in R^{C\times T}$, ECA first compresses each channel into a scalar descriptor through global average pooling, then captures local interactions among adjacent channels through a one-dimensional convolution with an adaptive kernel size, and ultimately generates channel weights through sigmoid activation. The computational process can be formulated as:

(4) $$s=\mathrm{GAP}\left(F\right)\in R^{C\times 1}$$

(5) $$w=\sigma {\left({\mathrm{Conv}1d}_{k}\left(s^{⊤}\right)\right)}^{⊤}\in R^{C\times 1}$$

(6) $$F^{\prime }=F⊙w$$

where GAP(·) denotes the global average pooling, σ(·) denotes the sigmoid activation function, ⊙ represents the channel-wise multiplication, and *k* represents the adaptive kernel size of the one-dimensional convolution. The kernel size *k* is adaptively determined based on the channel dimensionality *C* as:

(7) $$k={\left|\frac{\log_{2}C}{\gamma }+\frac{b}{\gamma }\right|}_{\mathrm{odd}}$$

where γ and *b* are hyperparameters that control the mapping relationship between channel dimensionality and interaction range, and ${\left|\;\cdot \;\right|}_{\mathrm{odd}}$ denotes rounding to the nearest odd integer.

### 2.6. Squeeze-And-Excitation Attention

**Figure 3:**
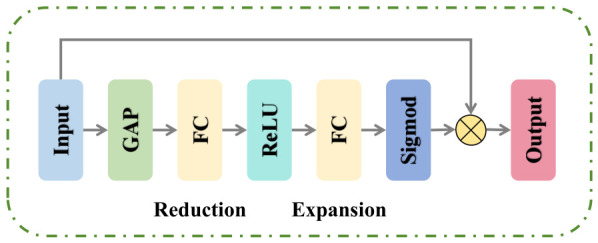
The principle of the SE attention mechanism.

SE is a channel attention mechanism proposed by Hu et al. [82], which achieves channel recalibration through two steps, namely global information squeeze and channel excitation. In RDAMNet, the complementary multi-scale features extracted by the three branches are concatenated along the channel dimension and compressed through a 1×1 convolution, after which the channels originating from different branches contribute differently to the final damage recognition. SE attention is introduced at the fusion stage to adaptively adjust the importance weights of each channel, thereby highlighting the channel responses most relevant to damage recognition in the fused features. The principle of SE attention is illustrated in Figure 3.

Given the fused feature $G\in R^{C^{\prime }\times T^{\prime }}$, SE first compresses the temporal information of each channel into a scalar descriptor through global average pooling, then learns the nonlinear inter-channel dependencies through a two-layer fully connected network, and ultimately generates a channel weight vector. The computational process can be formulated as:

(8) $$z=\mathrm{GAP}\left(G\right)\in R^{C^{\prime }}$$

(9) $$w_{\mathrm{SE}}=\sigma \left(W_{2}\;\cdot \;\mathrm{ReLU}\left(W_{1}\;\cdot \;z\right)\right)\in R^{C^{\prime }}$$

(10) $$G^{\prime }=G⊙w_{\mathrm{SE}}$$

where $W_{1}\in R^{\left(C^{\prime }/r\right)\times C^{\prime }}$ and $W_{2}\in R^{C^{\prime }\times \left(C^{\prime }/r\right)}$ are the learnable weight matrices of the two fully connected layers, respectively, *r* denotes the reduction ratio that controls the bottleneck dimensionality, and σ(·) represents the sigmoid activation function.

### 2.7. Adaptive Feature Fusion and Damage Recognition

After the feature extraction through the three branches described above, the output features $f_{1},f_{2},f_{3}\in R^{128\times T^{\prime }}$ from each branch are first aligned along the temporal dimension and then concatenated along the channel dimension to generate a joint feature representation. Subsequently, a 1×1 convolution compresses the channel dimensionality of the concatenated features from 3×128 to 128, reducing redundancy while achieving nonlinear cross-branch feature fusion. After the fused features undergo SE attention-based channel recalibration, two complementary global feature vectors are generated through global average pooling (GAP) and global max pooling (GMP), respectively. GAP captures the average activation response across each channel, reflecting the overall feature distribution. GMP captures the peak activation response across each channel, preserving the most salient discriminative information. The two global vectors are concatenated and subsequently processed through Dropout regularization and a two-layer fully connected network, ultimately yielding a *C*-dimensional damage recognition result vector. The fusion and damage recognition process can be formulated as:

(11) $$F_{\mathrm{cat}}=\mathrm{Concat}\left(f_{1},f_{2},f_{3}\right)\in R^{384\times T^{\prime }}$$

(12) $$F_{\mathrm{fuse}}=\mathrm{SE}\left(\mathrm{ReLU}\left(\mathrm{BN}\left({\mathrm{Conv}}_{1\times 1}\left(F_{\mathrm{cat}}\right)\right)\right)\right)\in R^{128\times T^{\prime }}$$

(13) $$v=\mathrm{Concat}\left(\mathrm{GAP}\left(F_{\mathrm{fuse}}\right),\mathrm{GMP}\left(F_{\mathrm{fuse}}\right)\right)\in R^{256}$$

(14) $$\hat{y}={\mathrm{FC}}_{2}\left(\mathrm{Dropout}\left(\mathrm{ReLU}\left({\mathrm{FC}}_{1}\left(\mathrm{Dropout}\left(v\right)\right)\right)\right)\right)\in R^{C}$$

where $\hat{y}$ denotes the predicted logit vector, and the final damage recognition result is obtained by selecting the damage pattern with the maximum predicted value.

## 3. Experimental Validation

### 3.1. Dataset Description

**Figure 4:**
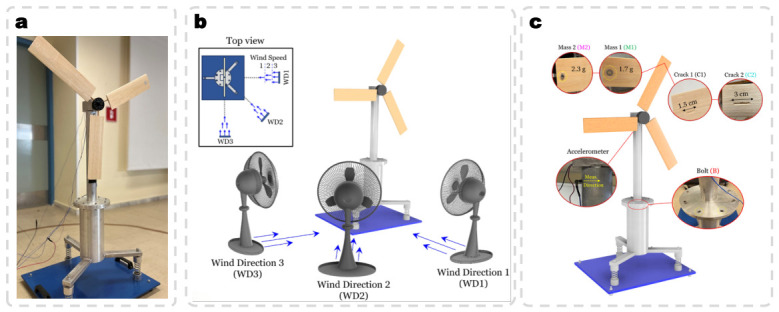
Experimental setup of the lab-scale floating wind turbine FWT. (**a**) Photograph of the lab-scale FWT model. (**b**) Schematic of the FWT under different wind directions WD1, WD2, and WD3, as well as different wind speeds WS1, WS2, and WS3. (**c**) Configuration of the lab-scale FWT model, including the accelerometer installation position and the considered damage scenarios. These scenarios consist of connection degradation between the tower and floater denoted as B, added mass cases where M1 corresponds to a mass of 1.7 g and M2 corresponds to a mass of 2.3 g, and blade crack cases where C1 corresponds to a crack length of 1.5 cm representing 4 percent of the blade length and C2 corresponds to a crack length of 3 cm representing 8 percent of the blade length.

The vibration data employed in this study are sourced from the UPATRAS Floating Wind Turbine Vibration Dataset [18], publicly released by the Stochastic Mechanical Systems and Automation Laboratory at the University of Patras, Greece. As illustrated in Figure 4, This dataset is based on a lab-scale FWT model, with vibration signals acquired by a single uniaxial accelerometer mounted on the upper part of the tower at a sampling frequency of $f_{s}=1024$ Hz. The dataset encompasses six structural health states, including one healthy state and five early-stage damage scenarios. The experiments were conducted under nine operating conditions derived from three wind speeds and three wind directions, yielding a total of 540 vibration signals.

This study selects the vibration signals of all six structural health states under the operating condition of Wind Direction 1 and Wind Speed 1, designated as WD1_WS1, as experimental data. The six structural health states and their corresponding labels are described as follows. The healthy state H indicates that the FWT structure is free from any artificial damage, serving as the baseline reference. The bolt connection degradation state B represents the connection degradation between the tower and floater, implemented by removing two of the eight mounting bolts. The added mass state M1 simulates the additional loading caused by ice accumulation by attaching a mass of 1.7 g to the blade edge. The added mass state M2 simulates more severe ice accumulation by attaching a larger mass of 2.3 g. The blade crack state C1 has a crack length of 1.5 cm, corresponding to approximately 4% of the overall blade length. The blade crack state C2 has a crack length of 3 cm, corresponding to approximately 8% of the overall blade length.

To ensure that no information leakage exists between the training and testing sets, a strict data partitioning strategy is adopted. First, the complete vibration recordings corresponding to each health state are divided into non-overlapping training and testing subsets at a ratio of 8:2. Subsequently, a non-overlapping sliding window of length 1024 is independently applied within the already-assigned training and testing subsets to segment the signals. Since the segmentation is performed strictly after the partitioning and no overlap exists between adjacent windows, the samples in the training and testing sets are completely isolated in time, eliminating any cross-contamination from adjacent or identical signal segments. The final dataset comprises 1140 training samples and 282 testing samples, yielding a total of 1422 samples.

### 3.2. Evaluation Metrics

Two evaluation metrics, damage recognition accuracy and weighted F1-score, are employed to evaluate the performance of the proposed method.

The damage recognition accuracy measures the overall recognition correctness of the model across all test samples, which is computed as

(15) $$\mathrm{Accuracy}=\frac{\mathrm{TP}+\mathrm{TN}}{\mathrm{TP}+\mathrm{TN}+\mathrm{FP}+\mathrm{FN}}\times 100\%$$

where TP, TN, FP, and FN denote the number of true positives, true negatives, false positives, and false negatives, respectively.

The weighted F1-score comprehensively considers the precision and recall of each damage pattern, providing a more thorough reflection of the model’s recognition performance under imbalanced conditions. For the multi-pattern damage recognition task, the weighted F1-score is computed as

(16) $${F1}_{\mathrm{weighted}}=\sum_{i=1}^{C}\frac{n_{i}}{N}\;\cdot \;\frac{2\;\cdot \;{\mathrm{Precision}}_{i}\;\cdot \;{\mathrm{Recall}}_{i}}{{\mathrm{Precision}}_{i}+{\mathrm{Recall}}_{i}}$$

where *C* denotes the total number of damage patterns, $n_{i}$ represents the number of samples of the *i*-th damage pattern, *N* denotes the total number of samples, and ${\mathrm{Precision}}_{i}$ and ${\mathrm{Recall}}_{i}$ represent the precision and recall of the *i*-th damage pattern, respectively.

### 3.3. Compared Methods

To comprehensively evaluate the performance of the proposed RDAMNet, five representative deep learning methods are selected as comparative baselines. These methods encompass different technical routes, including classical convolutional networks, multi-scale feature extraction, attention mechanism fusion, and spatiotemporal modeling, enabling the validation of RDAMNet from multiple perspectives. Each compared method is briefly introduced as follows.

1. ResNet18 [86] is a classical deep residual network based on convolutional neural networks, which effectively mitigates the gradient degradation problem in deep networks through the introduction of a residual learning framework. This method serves as a widely adopted baseline model in the deep learning community and is selected to verify the performance advantage of RDAMNet over general-purpose deep feature extraction architectures.
2. DCNet [87] is a dual-channel feature aggregation network proposed by Guo et al. for wind turbine fault diagnosis under variable speed operating conditions. This method constructs a parallel patch-aware convolutional module to extract multi-scale features from time-frequency representations, introduces Haar wavelet downsampling to reduce spatial resolution while preserving discriminative features, and dynamically allocates channel and spatial attention weights through a channel prior convolutional attention mechanism. This method is selected to evaluate the competitiveness of RDAMNet in attention mechanism-driven multi-scale feature fusion.
3. IMCTN [78] is a physics-aware spatiotemporal diagnostic framework proposed by Zhao et al. for structural health monitoring of ultra-large wind turbine blades. This method integrates ensemble empirical mode decomposition with a hybrid Transformer-CNN architecture, coupling multi-head self-attention with multi-scale convolutions to model long-range temporal dependencies and localized patterns. This method is selected to evaluate whether RDAMNet can achieve competitive damage recognition performance without the global modeling capability of Transformers.
4. MCAMCNN [88] is a fault diagnosis method based on a multi-channel attention mechanism convolutional neural network, proposed by Zheng et al. for wind turbine condition monitoring. This method employs a dual-layer multi-scale convolution combined with multi-channel attention to extract multi-domain features and dynamically calibrate feature channel weights, with adaptive feature fusion ultimately achieved through ECA. This method is selected to evaluate the performance difference between the dual attention mechanism of RDAMNet and the multi-channel attention strategy of this method in channel feature modeling.
5. MSCNN-BiLSTM-WMV [89] is a fusion model of multi-scale convolutional neural network and bidirectional long short-term memory network, proposed by Xu et al. for wind turbine bearing fault diagnosis. This method extracts spatial features through multi-scale convolutions, captures temporal dependencies through bidirectional LSTM, and proposes a weighted majority voting rule to fuse multi-sensor information for improving generalization capability. This method is selected to evaluate the effectiveness of the pure convolutional multi-branch architecture of RDAMNet compared with CNN-RNN hybrid architectures in temporal feature modeling.

### 3.4. Implementation Details

All experiments were conducted on a computer equipped with an Intel Core i9-14900KF processor, 64 GB RAM, and an NVIDIA GeForce RTX 5080 GPU. The network is implemented with Python 3.9.19 and PyTorch 2.0.0 framework. To ensure the fairness of comparative experiments, all compared methods use the same training and testing data split, receive the same vibration signals as input, and are trained and evaluated on the same hardware platform. Table 3 lists the detailed training configurations of all methods.

### 3.5. Signal-Level Motivation for Multi-Scale Modeling

**Figure 5:**
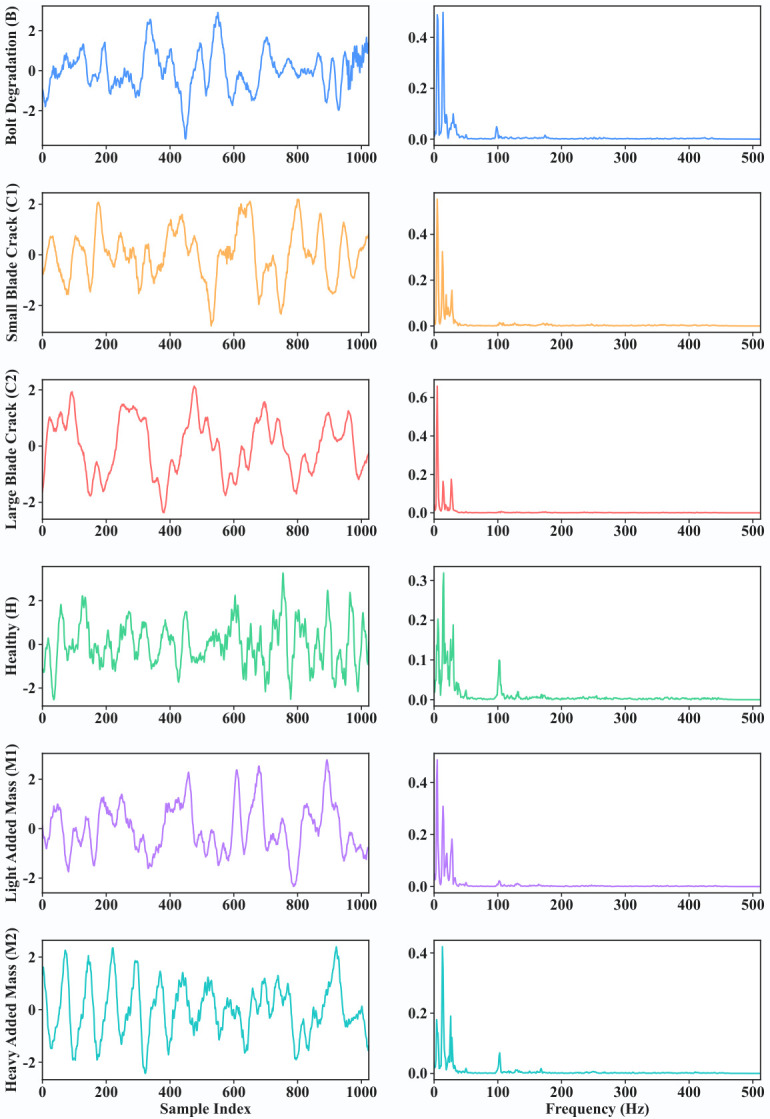
Time-domain and frequency-domain responses of randomly selected signal segments under different damage modes.

**Figure 6:**
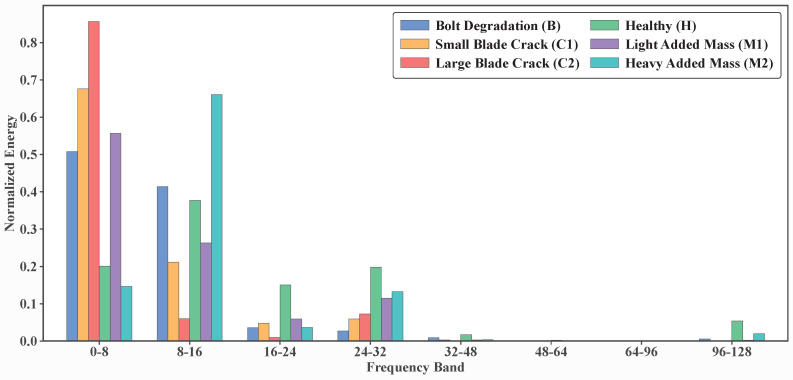
Normalized frequency-band energy distributions of different damage modes.

This subsection further analyzes the rationality of the multi-scale structure adopted in RDAMNet from the perspective of raw vibration signals, demonstrating that the proposed network design is derived from the intrinsic feature distribution of FWT damage-induced vibration responses rather than a simple stacking of architectural components. From the perspective of structural dynamics, the vibration response of an FWT results from the coupled interaction of wind loading, wave excitation, structural stiffness, and mass distribution. Different types of structural damage alter the system’s stiffness matrix, mass matrix, or damping properties, inducing multi-level dynamic effects such as natural frequency shifts, modal shape distortions, and transient response variations. These effects manifest as feature changes across different temporal scales in the vibration signals: variations in global stiffness or mass distribution predominantly affect the response characteristics of the dominant low-frequency modes, whereas localized structural defects tend to produce high-frequency transient components or local waveform distortions at shorter temporal scales. The time-domain waveforms and frequency-domain amplitude spectra of randomly selected signal segments under different damage modes are presented in Figure 5, and the normalized band energy distribution in the low-frequency range is presented in Figure 6.

As shown in Figure 5, all six structural states exhibit evident non-stationary vibration responses, whereas their amplitude envelopes, local peak distributions, and oscillatory patterns differ substantially. Bolt degradation state B produces pronounced amplitude fluctuations and local abrupt responses, which can be attributed to the reduction in tower-floater connection stiffness caused by the removal of mounting bolts, inducing larger relative displacements and nonlinear contact responses under dynamic loading. Blade crack states C1 and C2 exhibit varying degrees of oscillation intensity differences. The stiffness discontinuity introduced by cracks causes the cracked region to exhibit a breathing effect under alternating loads, thereby introducing localized nonlinear transient components into the time-domain signal. Added-mass states M1 and M2 present more evident low-frequency modulation, as the additional blade mass alters the mass distribution and moment of inertia of the rotating components, causing a downward shift of the structural natural frequencies. These phenomena indicate that damage-sensitive information is not concentrated at a single temporal scale, but is simultaneously distributed across global low-frequency trends, local peak responses, and medium-scale waveform variations.

The frequency-domain analysis further corroborates the above observation. Although the dominant vibration energy is concentrated in the low-frequency range, different damage modes exhibit distinct spectral peak locations, peak intensities, and band-energy distributions. The band-energy results in Figure 6 reveal that the relative energy contributions of different damage modes vary across low-frequency sub-bands, indicating that the diagnostic information encompasses both dominant low-frequency structural responses and weaker band-specific components with discriminative capability. A model employing only a single receptive field for feature extraction may overly focus on the dominant low-frequency energy, thereby insufficiently capturing weaker but critical damage-sensitive features in other time-frequency regions. RDAMNet addresses this issue through the synergy of its multi-branch differentiated architecture and dual attention mechanism. Specifically, the raw signal branch preserves the full time-domain resolution, and its convolutional kernels can directly operate on the original waveform containing high-frequency transient components. The max-pooled branch highlights transient impulses and abrupt variations by retaining local peak responses, which inherently correspond to the high-frequency energy components in the signal. Furthermore, the ECA attention embedded within each branch adaptively enhances damage-sensitive channel responses at each hierarchical level, preventing weaker but discriminative high-frequency feature channels from being overwhelmed by low-frequency dominant channels. After the features from the three branches are fused, SE attention further performs adaptive recalibration across cross-branch channels, ensuring that complementary features from different frequency ranges are effectively utilized.

The above analysis reveals that FWT damage-induced vibration responses simultaneously contain dominant low-frequency components, local transient variations, and subtle differences across frequency bands, exhibiting evident multi-scale properties. Single-scale feature learning alone may result in insufficient feature representation, particularly overlooking weaker but damage-related components. The multi-scale design of RDAMNet is motivated by this signal-level feature distribution, aiming to obtain more comprehensive damage-sensitive representations from different scales, thereby providing a more discriminative feature basis for subsequent damage pattern recognition.

### 3.6. Hyperparameter Sensitivity Analysis

**Figure 7:**
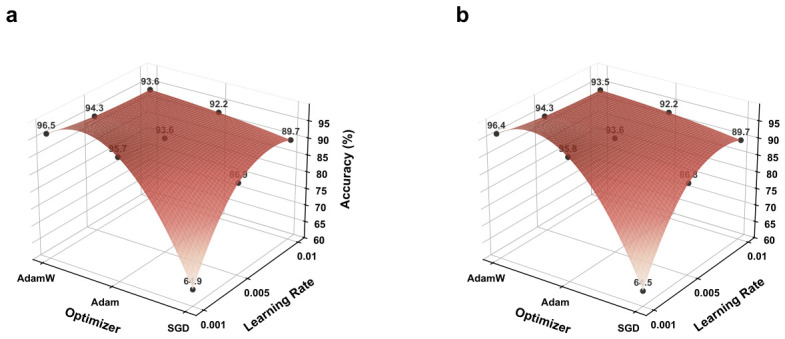
Hyperparameter sensitivity analysis of RDAMNet. (**a**) Accuracy surface across different optimizers and learning rates. (**b**) F1-score surface across different optimizers and learning rates.

To determine the optimal training configuration of RDAMNet, a joint sensitivity analysis is conducted on the optimizer and learning rate. Three optimizers, namely AdamW, Adam, and SGD, are combined with three learning rates of 0.001, 0.005, and 0.01 in a full factorial design, yielding 9 experiments in total. The results are presented as 3D surface plots in Figure 7. As shown in Figure 7, AdamW at a learning rate of 0.001 achieves the globally optimal performance, with the damage recognition accuracy and weighted F1-score reaching 96.45% and 96.44%, respectively. Both AdamW and Adam exhibit a monotonic performance decline as the learning rate increases, whereas SGD shows the opposite trend, achieving only 64.89% accuracy at a learning rate of 0.001 but improving to 89.71% at 0.01, which still remains significantly lower than the best result of AdamW. Based on the above analysis, all subsequent comparative experiments and ablation experiments adopt the optimal configuration of the AdamW optimizer and a learning rate of 0.001.

### 3.7. Comparative Results and Analysis

**Figure 8:**
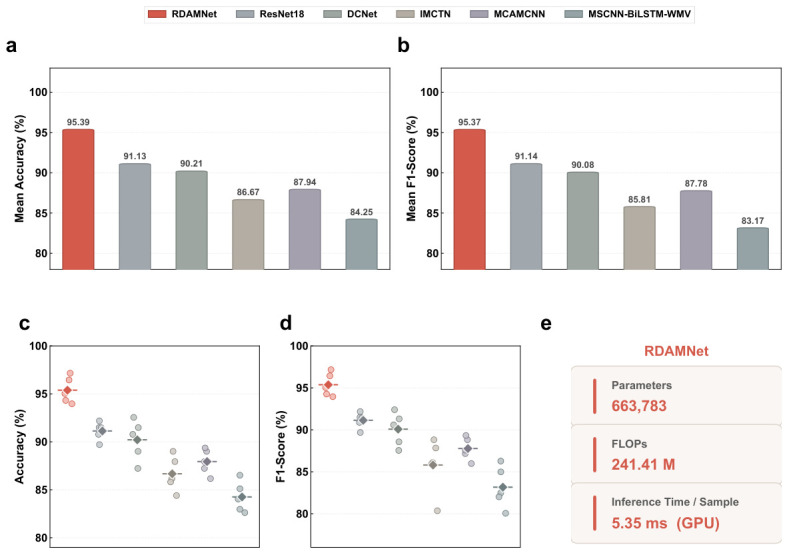
Comprehensive performance comparison of RDAMNet and baseline models. (**a**) Mean accuracy of six models across five independent runs. (**b**) Mean F1-score of six models across five independent runs. (**c**) Accuracy distribution over five runs. (**d**) F1-score distribution over five runs. (**e**) Model complexity of RDAMNet: parameters, FLOPs, and single-sample GPU inference time.

To comprehensively evaluate the robustness and stability of the models, all methods are independently run five times, each with a different random seed for data splitting and network parameter initialization, and results are reported as mean ± standard deviation. It should be noted that all the comparison results below were obtained on an independent test set. The comparative damage recognition results of all methods on the UPATRAS dataset are presented in Figure 8. As shown in Figure 8a,b, RDAMNet achieves the best mean damage recognition accuracy and weighted F1-score of 95.39 ± 1.23% and 95.37 ± 1.24%, respectively, across five independent runs, significantly outperforming all compared methods on both metrics. Compared with the classical ResNet18, RDAMNet demonstrates a substantial accuracy improvement. Although ResNet18 mitigates the gradient degradation problem in deep networks through residual connections, its single-scale feature extraction strategy fails to sufficiently capture the complementary information of multi-scale damage features in FWT vibration signals, resulting in insufficient discriminability for similar damage patterns. Compared with DCNet and MCAMCNN, which also employ attention mechanisms and multi-scale feature fusion, RDAMNet consistently maintains a clear performance lead. This performance difference is primarily attributed to the differentiated branch design of RDAMNet. Although DCNet and MCAMCNN introduce multi-scale convolutions and channel attention, both methods extract features from a unified input representation without achieving multi-scale decoupling at the signal level. RDAMNet generates complementary signals with different physical characteristics through max-pooling and average-pooling at the network front-end, enabling each branch to extract peak impulses, low-frequency structural responses, and long-range structural patterns under noise-suppressed conditions in a targeted manner, thereby obtaining richer discriminative feature representations. Furthermore, compared with the remaining baseline methods, RDAMNet also demonstrates significant performance advantages. This demonstrates that for the FWT vibration damage recognition task, the pure convolutional multi-branch architecture of RDAMNet combined with the dual attention mechanism achieves superior damage recognition performance without the need for complex temporal modeling components such as Transformers or recurrent neural networks.

Figure 8c,d further present the scatter distributions of accuracy and F1-score across five independent runs for each method. The scatter points of RDAMNet are concentrated in the high-performance region with relatively small fluctuations, indicating that it consistently achieves stable and high recognition performance under different random initializations. In contrast, some compared methods exhibit more dispersed distributions, reflecting their higher sensitivity to random seeds and insufficient stability. Furthermore, Figure 8e presents the model complexity metrics of RDAMNet. RDAMNet has only 663,783 parameters, 241.41 M FLOPs, and a single-sample GPU inference time of 5.35 ms, indicating that RDAMNet achieves the best recognition performance while maintaining low computational overhead, demonstrating a favorable performance-efficiency trade-off and practical deployment potential.

**Figure 9:**
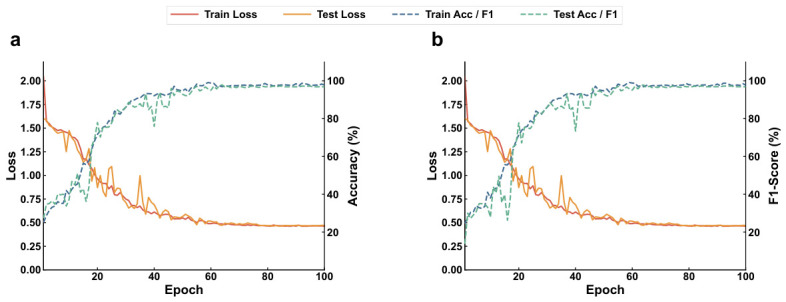
Training convergence and generalization of RDAMNet. (**a**) Training and testing loss and accuracy curves over 100 epochs. (**b**) Training and testing loss and F1-score curves over 100 epochs.

To further assess the overfitting risk of RDAMNet, Figure 9 simultaneously presents the training and testing loss, accuracy, and F1-score curves over 100 training epochs. The training and testing loss curves exhibit highly consistent trends, both showing steady decline and converging after approximately 60 epochs, with no typical overfitting signs such as the test loss increasing while the training loss continues to decrease. Meanwhile, the accuracy and F1-score of both the training and testing sets increase synchronously and converge to comparable levels, further confirming that RDAMNet possesses good generalization capability without significant overfitting.

**Figure 10:**
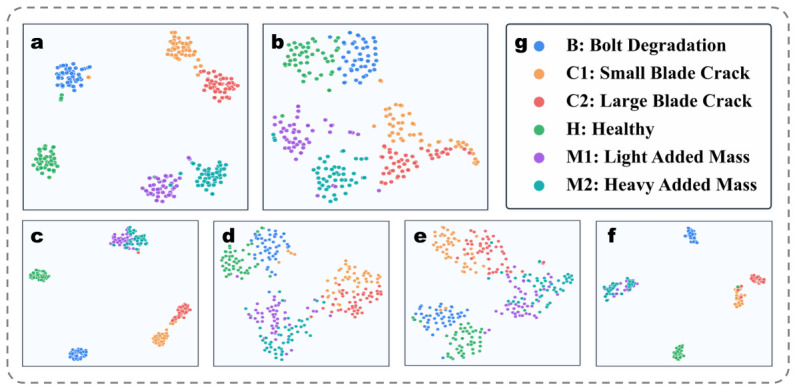
t-SNE visualization of the features extracted by different methods. (**a**) RDAMNet. (**b**) ResNet18. (**c**) DCNet. (**d**) IMCTN. (**e**) MCAMCNN. (**f**) MSCNN-BiLSTM-WMV. (**g**) Legend of damage pattern labels.

To further intuitively verify the above quantitative conclusions, t-SNE is employed to visualize the deep features extracted by each method, with the results presented in Figure 10, where subplot (g) annotates the damage pattern labels corresponding to different colors. As shown in Figure 10a, the features extracted by RDAMNet exhibit the most distinct clustering structure, with the six damage patterns forming tight and well-separated clusters in the feature space, and the inter-pattern boundaries are clearly distinguishable with virtually no cross-pattern sample overlap. As shown in Figure 10b, ResNet18 is capable of separating most damage patterns, yet overlap remains between some patterns. As shown in Figure 10c–f, the feature distributions of the remaining four compared methods all exhibit varying degrees of pattern overlap, with significant overlapping and intersection among the feature clusters of multiple damage patterns. The above visualization results corroborate the quantitative metrics presented in Figure 8, further verifying that RDAMNet achieves the optimal pattern separation in the feature space. This advantage stems from the synergistic effect of the multi-branch multi-scale feature extraction and the ECA-SE dual attention mechanism. The multi-scale branches capture complementary damage features from signals at different temporal resolutions, ECA enhances the responses of damage-sensitive channels within each branch, and SE adaptively recalibrates the contribution weights of cross-branch channels at the fusion stage. The three components collectively ensure that the final feature representation possesses high intra-pattern compactness and inter-pattern separability.

### 3.8. Cross-Condition Generalization Analysis

**Figure 11:**
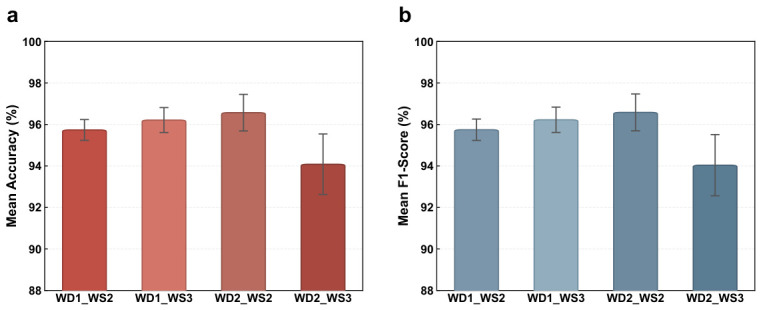
Performance of RDAMNet under different operating conditions. (**a**) Mean accuracy with error bars across three independent runs. (**b**) Mean F1-score with error bars across three independent runs.

The preceding comparative experiments validate the damage recognition performance of RDAMNet under the single operating condition of WD1_WS1. However, FWTs in practical operation face continuously varying wind speed and wind direction conditions, and the model’s generalization capability across different operating conditions is a critical prerequisite for its practical deployment. To this end, this subsection further evaluates the cross-condition generalization performance of RDAMNet under different wind speed and wind direction combinations. In addition to the training condition WD1_WS1, four different wind speed and wind direction combinations, namely WD1_WS2, WD1_WS3, WD2_WS2, and WD2_WS3, are selected. RDAMNet is independently trained and tested under each operating condition, with three independent runs per condition, and the results are presented in Figure 11.

As shown in Figure 11, the mean accuracy and mean F1-score of RDAMNet exceed 94% across all four operating conditions, with small standard deviations, indicating that the model maintains stable and high recognition performance under different wind speed and wind direction conditions. These results demonstrate that the damage-sensitive feature representations learned through the multi-branch multi-scale feature extraction strategy and dual attention mechanism exhibit favorable cross-condition transferability. It is noteworthy that the performance under WD2_WS3 is slightly lower than under other conditions, which may be attributed to the increased signal complexity and noise level caused by the coupled effects of higher wind speed and different wind direction. Nevertheless, the mean accuracy under this condition still exceeds 94%, indicating that RDAMNet remains capable of effectively recognizing damage patterns under more challenging operating conditions.

### 3.9. Ablation Study

To systematically verify the contribution of each core component of RDAMNet to the final damage recognition performance, six ablation experiments are designed in this subsection. All ablation experiments are conducted under the same training hyperparameters and data split settings as the comparative experiments above, ensuring fair comparability. The ablation variants are designed as follows:

1. **w/o Multi-scale Input:** The inputs of all three branches are unified to the raw signal by removing the max-pooling and average-pooling operations at the input stage, to verify the effectiveness of signal-level multi-scale decoupling.
2. **w/o Multi-branch:** Only the raw signal branch is retained, and the max-pooled branch and the average-pooled dilated convolution branch are removed, to verify the necessity of the multi-branch architecture.
3. **w/o ECA:** w/o ECA: The ECA channel attention module is removed from all ResECA blocks, to verify the role of intra-branch hierarchical channel attention.
4. **w/o SE:** The SE channel attention module is removed from the fusion layer, to verify the role of channel recalibration at the fusion stage.
5. **Dual Attention:** Both ECA and SE attention modules are simultaneously removed, to verify the overall synergistic effect of the dual attention mechanism.
6. **w/o Residual:** The residual connections are removed from all ResECA blocks, to verify the role of residual connections in preserving subtle damage features.

The ablation study results are presented in Table 4. Among all ablation variants, w/o Multi-scale Input leads to the most significant performance degradation, with the accuracy dropping from 96.45% to 87.23%, indicating that signal-level multi-scale decoupling is the core driving factor of RDAMNet’s performance. The accuracy of w/o Multi-branch further drops to 89.36%, verifying the necessity of the multi-branch architecture in extracting complementary damage features. Regarding the attention mechanisms, the accuracies of w/o ECA and w/o SE decrease to 92.19% and 93.97%, respectively, while w/o Dual Attention, which simultaneously removes both modules, results in an accuracy of 91.13%, lower than that of removing either attention module alone. This indicates a significant synergistic gain between ECA and SE, where ECA hierarchically enhances the responses of damage-sensitive channels within each branch, and SE adaptively recalibrates the contribution weights of cross-branch channels at the fusion stage, thereby achieving a performance gain greater than the sum of their individual contributions. Additionally, w/o Residual yields an accuracy of 94.32%, verifying the role of residual connections in facilitating the propagation of subtle damage information during deep feature extraction. The above results collectively demonstrate that each core component in RDAMNet contributes indispensably to the final damage recognition performance, where multi-scale input decoupling and the multi-branch architecture constitute the performance foundation, the dual attention mechanism provides critical feature enhancement, and residual connections ensure the integrity of feature propagation.

Notably, the contributions of these components are not simply independent and additive. Multi-scale input decoupling provides each branch with complementary signal representations possessing distinct physical characteristics, upon which the multi-branch architecture extracts damage-sensitive features at different scales through differentiated kernel sizes and dilation rates. ECA then hierarchically suppresses noise-dominant channels and enhances damage-related channel responses within each branch, thereby ensuring that the branch-level features delivered to the fusion stage possess a high signal-to-noise ratio. Building on this, SE adaptively recalibrates channels according to their contributions to the classification objective, achieving effective integration of cross-branch complementary information, while residual connections span the entire feature extraction process to safeguard subtle damage features from being lost during layer-by-layer abstraction. This hierarchical cooperation also explains why w/o Dual Attention yields lower performance than removing either ECA or SE alone: when both attention mechanisms are simultaneously absent, the adaptive regulation of intra-branch feature purification and cross-branch feature integration is lost, resulting in the complete forfeiture of synergistic gains.

### 3.10. Interpretability Analysis of Multi-Scale Features

**Figure 12:**
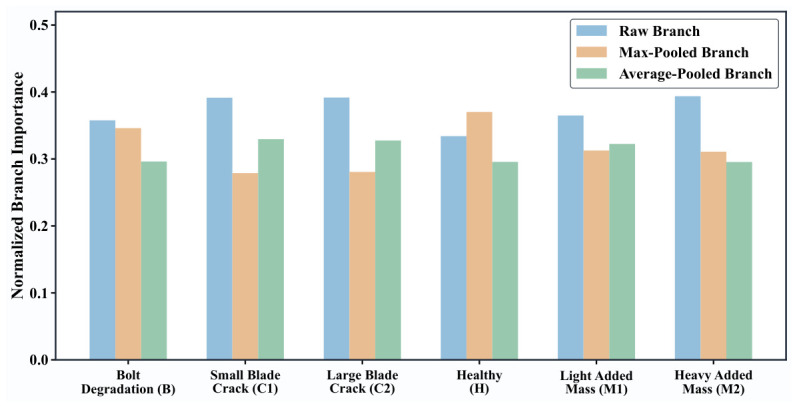
Branch importance analysis of the three RDAMNet branches under different damage modes.

**Figure 13:**
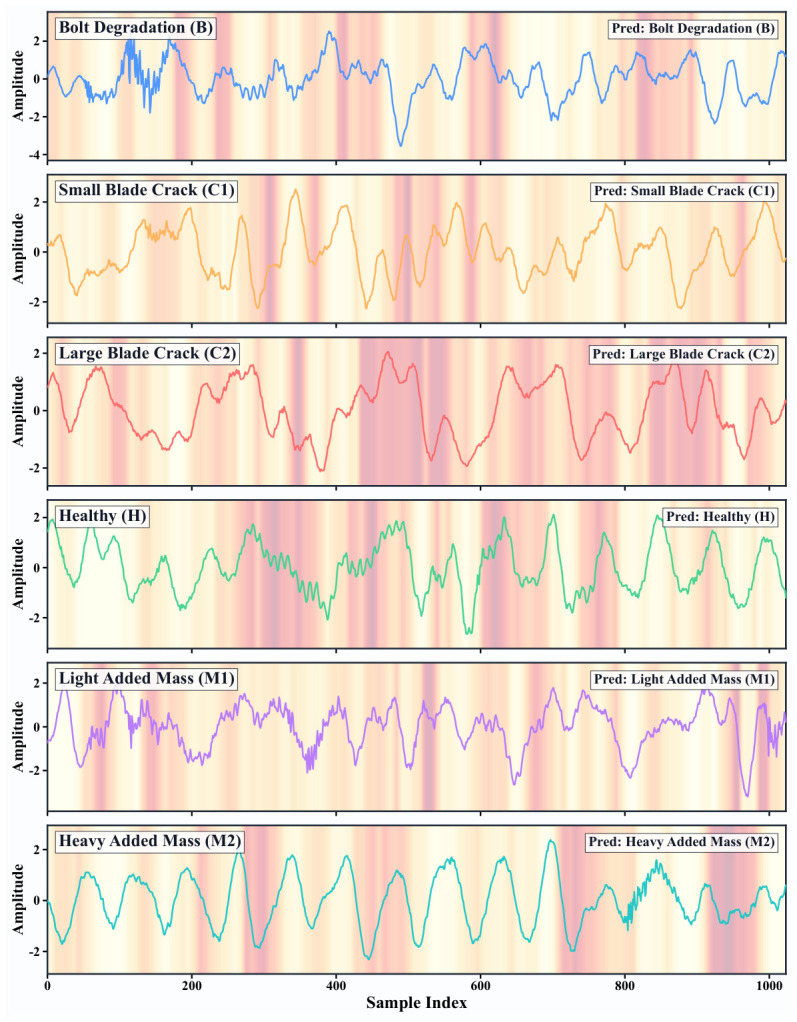
One-dimensional Grad-CAM visualization of RDAMNet under different damage modes.

To examine whether the trained RDAMNet captures discriminative representations at different temporal resolutions, branch importance analysis and one-dimensional Grad-CAM visualization are introduced based on Grad-CAM [90]. Grad-CAM is a gradient-based interpretability method that highlights the feature regions most relevant to the target-class prediction by backpropagating class-specific gradients. Following this principle, the branch importance is calculated by measuring the gradient magnitude of the target-class score with respect to the output feature map of each RDAMNet branch. The obtained values are then normalized by the total importance of all three branches, allowing a direct comparison among different health and damage states. The results are presented in Figure 12 and Figure 13, respectively. Figure 12 presents the normalized importance distribution of the three branches under different damage modes.

All three branches contribute to the final decision, but their relative importance varies with the damage mode. For blade crack C1, blade crack C2, and heavy added mass M2, the raw-signal branch shows the highest importance, indicating that local waveform details and transient variations at the original temporal resolution are highly informative for these cases. For the healthy state H, the max-pooled branch becomes dominant, suggesting that peak-preserved patterns are useful for identifying the baseline vibration behavior without artificial damage. For bolt degradation B, the raw-signal and max-pooled branches make comparable contributions, implying that this state contains both abrupt local responses and detailed waveform information. In contrast, the average-pooled branch provides relatively stable support across all damage modes, reflecting the complementary role of smoothed low-frequency structural responses.

The above branch importance distribution shows that different damage modes do not rely on a single-scale representation. Instead, RDAMNet adaptively emphasizes different branches according to the vibration characteristics of each condition. This result corroborates the ablation conclusions in Table 4, further confirming that multi-scale input decoupling and the multi-branch architecture are important sources of the performance improvement of RDAMNet. Figure 13 further presents the one-dimensional Grad-CAM responses under different damage modes. All six damage mode samples are correctly recognized, and the high-response regions are distributed across multiple temporal segments rather than concentrated at isolated sampling points. For blade crack states, the activation regions predominantly appear at positions with enhanced local oscillations and evident waveform variations. For added-mass states, especially M2, the activation responses extend across several low-frequency oscillation cycles. For bolt degradation, the high-response regions are associated with local peak-valley variations and non-stationary fluctuations. These results demonstrate that RDAMNet simultaneously attends to local transient features, waveform details, and longer-range structural response variations during damage recognition.

Overall, the branch importance analysis and Grad-CAM visualization provide interpretability evidence that the three branches form a complementary feature extraction system rather than redundantly learning similar information. The raw-signal branch captures fine-grained transient details, the max-pooled branch emphasizes peak-related patterns, and the average-pooled branch supplies a noise-suppressed low-frequency baseline. Moreover, the dispersed Grad-CAM activations indicate that the predictions are mainly supported by structural vibration patterns over continuous temporal regions, instead of by isolated noise-like points. This improves the physical credibility of RDAMNet and further explains its effectiveness in FWT vibration damage recognition.

### 3.11. Engineering Implications for Condition-Based Maintenance

The experimental results indicate that RDAMNet can distinguish the six structural health states of FWTs with high reliability, providing a technical basis for condition-based maintenance in offshore wind farm operation. In engineering practice, different fault patterns imply different risk levels and intervention priorities. Bolt connection degradation B represents the loosening or deterioration of fasteners between the tower and floater. If this condition is not detected and addressed in time, stress concentration may increase in the connection region, potentially accelerating fatigue crack initiation and threatening global structural stability. Therefore, this state should be regarded as a high-priority warning condition. Blade crack states C1 and C2 correspond to structural defects with different severity levels. Since cracks may continue to propagate under cyclic wind-wave loading, their timely identification allows maintenance personnel to plan targeted inspection or repair before the defect reaches a critical stage, thereby reducing the risk of blade fracture. Added-mass states M1 and M2 represent mass imbalance caused by ice accumulation. Such loading can change the dynamic characteristics of rotating components and increase the wear of bearings and drivetrain systems. Early recognition of these states can support timely de-icing decisions and prevent secondary mechanical deterioration.

From a decision-making perspective, RDAMNet goes beyond binary anomaly detection by further identifying the fault category and severity level. This capability enables graded maintenance responses. For example, a minor blade crack C1 may be incorporated into the next planned inspection cycle, whereas a more severe crack C2 or bolt degradation B should trigger a higher-priority intervention. Such a differentiated strategy can help balance structural safety and maintenance cost by avoiding both unnecessary downtime caused by over-maintenance and risk accumulation caused by delayed repair.

This implication is particularly important for FWTs deployed in deep-sea environments, where offshore maintenance is strongly constrained by weather windows, vessel availability, accessibility, and personnel safety. Conventional time-based preventive maintenance cannot fully reflect the actual structural condition and may lead to inefficient inspections or delayed responses. As an automated vibration-based recognition model, RDAMNet can be integrated into an online monitoring framework using vibration signals continuously collected by nacelle-mounted accelerometers. In this way, the model can provide real-time or near-real-time health assessment for maintenance planning. In addition, its compact model size and millisecond-level inference speed support edge-side deployment, which is beneficial for reducing data transmission burden and enabling fast local diagnosis. These characteristics make RDAMNet a promising tool for supporting the transition from passive periodic inspection to proactive health-aware maintenance, with potential benefits in reducing unplanned downtime, extending structural service life, and improving the economic efficiency of offshore wind farm operation.

### 3.12. Limitations and Future Work

Although RDAMNet achieves strong performance on the laboratory dataset, several limitations should be acknowledged. First, the UPATRAS dataset is obtained from a laboratory-scale FWT model, whose dynamic behavior may differ from that of full-scale offshore turbines. Real marine environments involve more complex disturbances, including sensor noise, non-stationary wind-wave excitations, and long-term signal drift, which may affect recognition accuracy. In addition, the structural scale, material properties, boundary conditions, and damage morphologies of practical turbines are more complicated than those represented in the experimental model. Therefore, the transferability of RDAMNet to real offshore structures still requires further validation. Second, this study considers six discrete health or damage states, while actual faults usually evolve continuously and may appear as compound scenarios involving multiple simultaneous defects. Third, although the cross-condition experiments show stable performance under different wind speed and direction combinations, real offshore operating conditions are broader and more extreme. The robustness of the model under severe sea states, storms, surges, and long-term operational uncertainty remains to be further investigated.

Future work will focus on validating RDAMNet using field monitoring data from real offshore wind farms and improving its adaptability to industrial environments. Domain adaptation and transfer learning can be introduced to reduce the distribution gap between laboratory and field data. Robust recognition under missing data, sensor degradation, and variable noise levels also deserves further study. In addition, the damage categories should be extended from discrete single-fault states to continuous degradation processes and multi-fault coupling conditions. Another important direction is to combine fault recognition with long-term degradation modeling and remaining useful life prediction, so that the framework can be further developed from state classification toward structural life-cycle assessment and maintenance optimization.

## 4. Conclusions

This paper proposes RDAMNet, a Residual Dual Attention Multiscale Network for vibration-based damage recognition of floating wind turbines. RDAMNet introduces a novel multi-branch multi-scale input strategy that decouples the raw vibration signal at the signal level into complementary representations with distinct physical characteristics, enabling different branches to capture damage-sensitive features at different scales. Additionally, an ECA-SE dual attention mechanism is designed to collaboratively enhance damage-related information at both the feature extraction and fusion stages. On the UPATRAS Floating Wind Turbine Vibration Dataset, RDAMNet achieves a mean damage recognition accuracy and a weighted F1-score of 95.39 ± 1.23% and 95.37 ± 1.24%, respectively, across five independent runs, significantly outperforming five representative methods including ResNet18, DCNet, IMCTN, MCAMCNN, and MSCNN-BiLSTM-WMV. Cross-condition generalization experiments demonstrate that RDAMNet maintains mean accuracies exceeding 94% across different wind speed and wind direction combinations, validating its generalization capability under different operating conditions. Model complexity analysis reveals that RDAMNet contains only 663,783 parameters, 241.41 M FLOPs, and a single-sample GPU inference time of 5.35 ms, achieving a balance between recognition performance and computational efficiency. The ablation study demonstrates that each core component contributes indispensably to the recognition performance, with multi-scale input decoupling and the multi-branch architecture serving as the performance foundation, while the dual attention mechanism provides critical feature enhancement. Branch importance analysis and Grad-CAM visualization further confirm that RDAMNet adaptively leverages features at different scales according to the vibration response characteristics of distinct damage modes. These results demonstrate that RDAMNet provides an effective and interpretable solution for vibration-based damage recognition in the structural health monitoring of floating wind turbines.

## Figures and Tables

**Table 1:** Detailed architecture of RDAMNet.

Module	Layer	Filters	Kernel/Dilation	Output
Branch 1 (Raw)	ResECA × 3	32→64→128	49/1, 15/1, 7/1	128×128
Branch 2 (MaxPool)	ResECA × 3	32→64→128	9/1, 5/1, 3/1	128×128
Branch 3 (AvgPool)	ResECA × 3	32→64→128	7/2, 5/2, 3/4	128×128
Fusion	Conv 1×1 + SE	384→128	–	128×128
Classifier	GAP + GMP + FC	256→128→C	–	*C*

**Table 2:** Block specifications of ResECA, ECA, and SE modules.

Block	Parameter	Value
ResECA	Conv layers per block	2
ResECA	Shortcut	Identity/1 × 1 Conv + BN
ResECA	Temporal downsampling	MaxPool1d, stride 2
ECA	γ, *b*	2, 1
SE	Reduction ratio *r*	8

**Table 3:** Training configurations of all compared methods.

Method	Optimizer	Learning Rate	Batch Size	Epochs	Loss Function
RDAMNet	AdamW	0.001	64	100	CE (label smoothing = 0.1)
ResNet18	AdamW	0.001	64	150	CE (label smoothing = 0.1)
DCNet	Adam	0.001	32	50	CE
IMCTN	AdamW	0.001	64	50	CE
MCAMCNN	Adam	0.01	16	100	CE
MSCNN-BiLSTM-WMV	Adam	0.001	64	100	CE

**Table 4:** Ablation study results of RDAMNet on the UPATRAS dataset.

Variant	Accuracy (%)	F1-Score (%)
RDAMNet	**96.45**	**96.44**
w/o Multi-scale Input	87.23	87.02
w/o Multi-branch	89.36	89.31
w/o ECA	92.19	92.16
w/o SE	93.97	93.27
w/o Dual Attention	91.13	91.14
w/o Residual	94.32	94.28

## Data Availability

Data are available on request from the corresponding authors.
